# Structural and Functional Characteristics of the Liver After Fractionated Local Electron Irradiation and Against the Background of Ascorbic Acid Administration

**DOI:** 10.3390/jpm16090439

**Published:** 2026-08-22

**Authors:** Grigory Demyashkin, Anastasiia Buianova, Nathalia Pyatigorskaya, Olga Filippova, Galina Brkich, Zhanna Aladysheva, Vasiliy Belyaev, Vladimir Shchekin

**Affiliations:** 1 Department of Industrial Pharmacy, Institute of Professional Education, I.M. Sechenov First Moscow State Medical University (Sechenov University), Trubetskaya St., 8/2, Moscow 119048, Russia; pyatigorskaya_n_v@staff.sechenov.ru (N.P.); ffiona@mail.ru (O.F.); brkich_g_e@staff.sechenov.ru (G.B.); aladysheva_zh_i@staff.sechenov.ru (Z.A.); belyaev_v_v_1@staff.sechenov.ru (V.B.); 2 Research and Educational Resource Center for Immunophenotyping, Digital Spatial Profiling and Ultrastructural Analysis Innovative Technologies, Peoples’ Friendship University of Russia, Miklukho-Maklaya, 6, Moscow 117198, Russia; anastasiiabuianova97@gmail.com (A.B.); dr.shchekin@mail.ru (V.S.)

**Keywords:** radiation-induced liver disease, electron irradiation, beta particles, radioprotection, ascorbic acid, oxidative stress, hepatic fibrosis

## Abstract

Radiation-induced liver disease (RILD) remains a clinically significant complication of radiotherapy for malignant neoplasms of the liver and upper abdomen, restricting achievable therapeutic doses and adversely affecting patient outcomes. Conventional photon-based radiotherapy (X-rays/γ-rays) exposes substantial volumes of healthy liver parenchyma and adjacent organs to ionizing radiation, increasing the risk of hepatocellular damage, inflammation, and progressive fibrosis. Electron irradiation (β-particles) represents a promising alternative owing to its limited tissue penetration (~2–3 cm) and steep dose fall-off, which significantly reduces exit dose and may reduce exit dose and limit exposure of paratumoral healthy tissues compared with photon therapy. However, the molecular mechanisms underlying electron-induced hepatic damage and strategies for its prevention remain insufficiently characterized. This review systematically analyzes the pathogenesis of radiation-induced liver damage, encompassing direct DNA damage, oxidative stress, mitochondrial dysfunction, NF-κB-mediated inflammation, cellular senescence, and TGF-β/Smad-driven fibrosis. The comparative dosimetric and radiobiological advantages of electron irradiation over photon therapy are discussed, with particular attention to intraoperative radiotherapy, FLASH, and very high-energy electron techniques. Current radioprotective strategies are critically evaluated, with emphasis on the limitations of amifostine and the multifunctional radioprotective potential of ascorbic acid, including free radical scavenging, attenuation of lipid peroxidation, stimulation of endogenous antioxidant defense, and direct inhibition of radiation-induced DNA strand breaks. Existing gaps in the evidence base are identified, and recommendations for future experimental and clinical research are proposed.

## 1. Introduction

Radiotherapy (RT) is one of the principal treatment modalities for malignant neoplasms of the liver and upper abdomen, employed as a definitive, adjuvant, neoadjuvant, or palliative intervention [1,2]. However, ionizing radiation (IR) inevitably affects not only atypical cells but also healthy tissues within the irradiation field, and the liver—one of the most radiosensitive organs—is particularly vulnerable [3]. Radiation-induced liver disease (RILD) develops in 5–10% of cases at doses of 30–35 Gy and in up to 50% of cases at 43 Gy [4,5], whereas effective tumor control for hepatocellular carcinoma (HCC) requires doses exceeding 60 Gy, a range associated with up to 76% mortality risk due to liver failure. Even single X-irradiation at 8 Gy causes morphological changes including edema, hemorrhage, and sinusoidal stasis, while total gamma irradiation at as low as 4 Gy induces DNA breaks, mitochondrial destruction, and hepatocyte apoptosis at the cellular and subcellular levels [6]. The use of RT in oncology is regulated by standard clinical protocols; however, measures for the prevention and treatment of acute and chronic radiation hepatitis remain insufficiently defined [7].

To date, the molecular mechanisms of X- and gamma radiation effects on the liver are relatively well characterized, encompassing DNA damage, oxidative stress, inflammation, and progressive fibrosis, leading to impaired metabolic and detoxification functions and, in severe cases, liver failure [8]. During RT for abdominal malignancies, healthy liver parenchyma and adjacent organs inevitably fall within the irradiation zone, necessitating strategies that minimize hepatic toxicity without compromising antitumor efficacy.

In this context, electron irradiation (β-particles) represents a promising alternative to conventional photon-based therapy. Due to their limited tissue penetration (~2–3 cm) and steep dose fall-off at practical range, electrons deliver minimal exit dose and substantially reduce exposure of paratumoral healthy tissues [9], making them particularly relevant for intraoperative RT of liver tumors and upper abdominal malignancies. However, qualitative and quantitative changes in liver structures under beta irradiation—including effects on hepatocyte proliferation, differentiation, apoptosis, and the endogenous redox system—remain poorly studied, and existing data are largely extrapolated from photon irradiation models.

Equally unresolved is the question of effective hepatic radioprotection. Among currently available agents, amifostine is the only broadly recommended radioprotector with proven efficacy; however, its clinical utility is limited by significant toxicity, a narrow therapeutic window, and the requirement for parenteral administration. Ascorbic acid represents a candidate alternative, combining high antioxidant activity, a wide therapeutic range, and minimal side effects. Beyond conventional free radical scavenging, it has been shown to stimulate endogenous antioxidant defense, reduce lipid peroxidation, and—uniquely among antioxidants—directly inhibit radiation-induced DNA strand breaks via copper ion chelation [10]. Nevertheless, comprehensive evidence for its radioprotective efficacy specifically in the liver under conditions of electron irradiation is lacking.

This review aims to answer four specific questions regarding radiation-induced liver damage. First, which electron modalities can physically reach liver targets given their limited penetration depth? Second, what dosimetric advantages and penalties arise when comparing electron versus photon therapy in this context? Third, what evidence supports electron-specific liver injury mechanisms? Fourth, what clinical or preclinical evidence supports ascorbic acid as a candidate radioprotective agent? By systematically addressing these questions, we identify current evidence gaps and propose future research directions.

## 2. Methods

A narrative literature review was conducted to evaluate the epidemiology, pathogenesis, molecular mechanisms, and potential prevention strategies of RILD, with particular emphasis on the comparative effects of photon and electron irradiation, oxidative stress-mediated hepatic damage, and the radioprotective properties of ascorbic acid.

Electronic databases including PubMed/MEDLINE, Scopus, and eLibrary were searched using combinations of Medical Subject Headings (MeSH) terms and free-text keywords related to the following domains: “radiation-induced liver disease,” “RILD,” “radiation-induced liver fibrosis,” “electron irradiation,” “hepatocellular carcinoma,” “oxidative stress,” “reactive oxygen species,” “lipid peroxidation,” “DNA damage,” “cellular senescence,” “hepatic stellate cells,” “radioprotection,” “amifostine,” “ascorbic acid,” “vitamin C,” and “bystander effect.” Boolean operators (AND, OR) were used to refine search strategies.

The review included experimental animal studies (rat and mouse models of hepatic irradiation), mechanistic molecular investigations (in vitro and in vivo), clinical studies of RILD in patients receiving radiotherapy for hepatobiliary and upper abdominal malignancies, clinical guidelines, and review articles addressing radiation-induced hepatic injury, fibrotic signaling pathways, inflammatory responses, and antioxidant-mediated radioprotection. Particular attention was given to studies investigating intracellular signaling pathways implicated in hepatocyte survival, hepatic stellate cell (HSC) activation, and radiation response, including ATM/ATR/p53, TGF-β/Smad, NF-κB, MAPK, JAK/STAT, and ferroptosis-associated mechanisms.

Reference lists of eligible publications were manually screened to identify additional relevant sources. Publications in English were prioritized. No strict publication date restrictions were applied. Nevertheless, priority was given to studies published from 2015 onward, which constituted the majority of the included literature, while seminal earlier studies were retained when they represented foundational evidence regarding radiation biology, RILD pathogenesis, hepatic fibrosis, apoptosis, or antioxidant mechanisms. Contemporary clinical guidelines (NCCN, 2026) were included to reflect current evidence-based recommendations for radiotherapy in hepatobiliary and upper abdominal malignancies.

As the present work represents a broad narrative synthesis intended to integrate experimental, translational, and clinical evidence on the pathophysiology of RILD and the potential of radioprotective strategies, no formal systematic review protocol, PRISMA framework, risk-of-bias assessment, or quantitative meta-analysis was performed.

**Figure 1:**
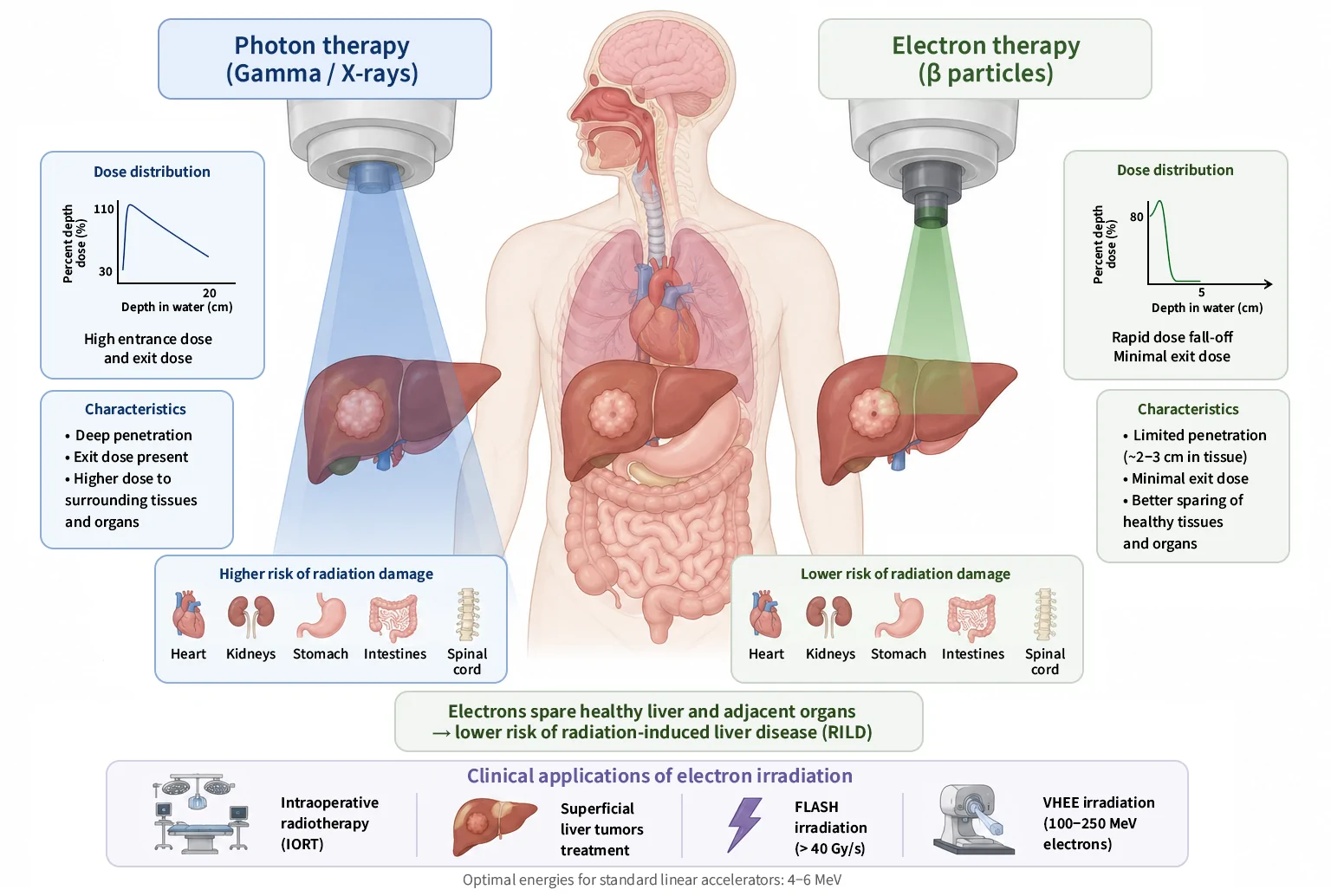
Comparison of ionizing radiation types for liver irradiation. Gamma/X-rays (photons) have high penetrating ability and deliver entrance and exit doses, exposing large volumes of healthy liver and adjacent organs, which increases the risk of radiation-induced liver disease (RILD). Electrons (β-particles) have limited penetration (≈2–3 cm in tissue) and exhibit a steep dose fall-off at practical range, resulting in minimal exit dose and better sparing of surrounding healthy tissues. Electrons are particularly promising for intraoperative radiotherapy (IORT), superficial liver tumors, and advanced techniques such as FLASH (ionising radiation is delivered at ultra-high dose rates—typically >40 Gy/s—over very short time scales) and VHEE (Very High-Energy Electron). Optimal energies for standard linear accelerators are 4–6 MeV.

**Figure 2:**
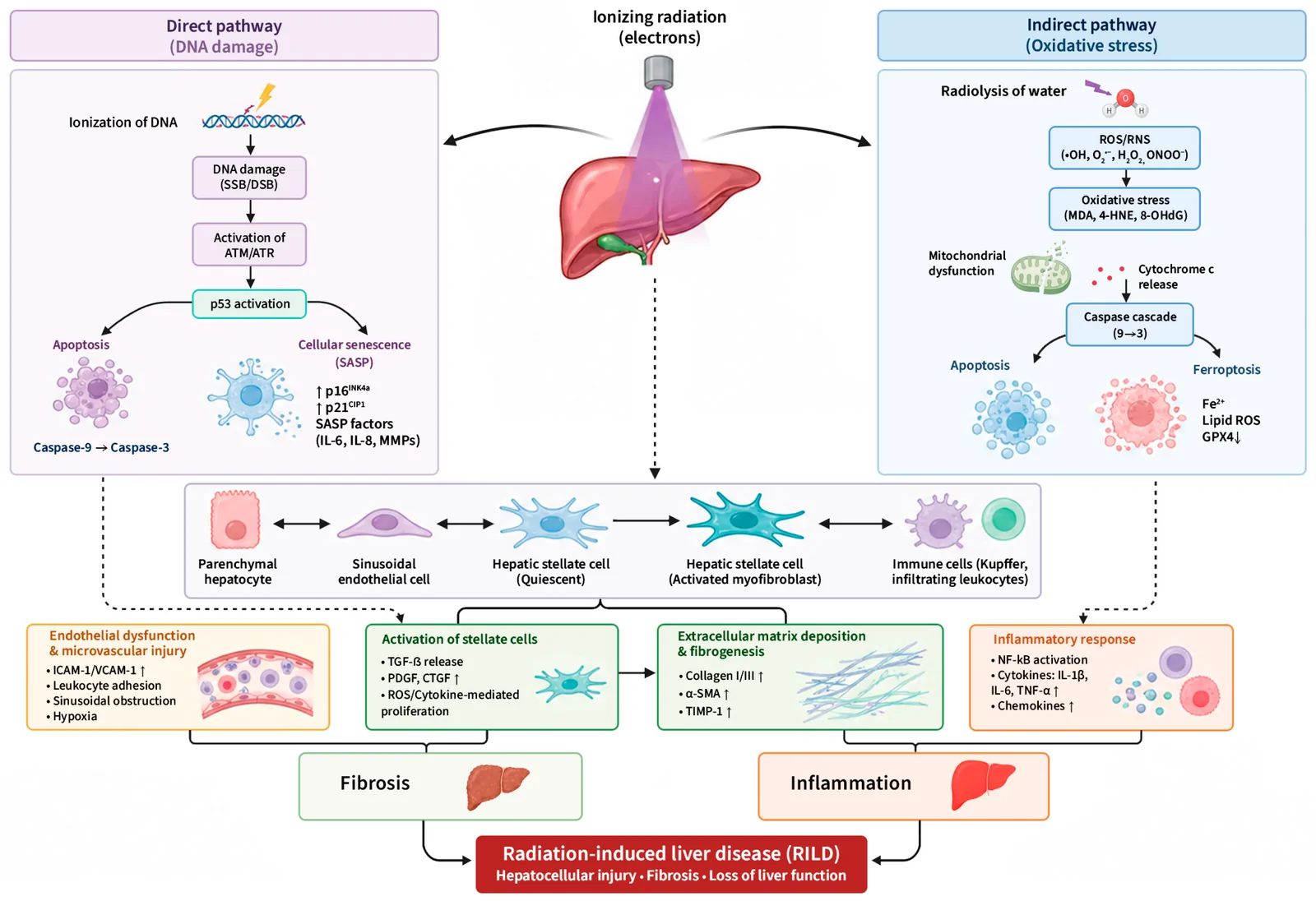
Pathogenesis of radiation-induced liver disease (RILD). Ionizing radiation (electrons) injures the liver through direct and indirect mechanisms. Direct DNA damage in hepatocytes activates the ATM/ATR-p53 pathway, leading to apoptosis or cellular senescence with a pro-inflammatory senescence-associated secretory phenotype (SASP). Indirect effects are mediated by radiolysis of water and the generation of reactive oxygen and nitrogen species (ROS/RNS), causing oxidative stress, mitochondrial dysfunction and activation of apoptotic and ferroptotic cell death pathways. Both mechanisms converge on sinusoidal endothelial cells, hepatic stellate cells and immune cells. Endothelial dysfunction promotes microvascular disturbances and hypoxia. Activation of hepatic stellate cells via profibrogenic mediators such as TGF-β drives extracellular matrix deposition and fibrosis. Activation of NF-κB and the release of pro-inflammatory cytokines amplify inflammatory responses. The integrated effects of fibrosis, inflammation and hepatocellular loss culminate in radiation-induced liver disease.

Figure 1 and Figure 2 were partly generated using ChatGPT-5.5 (OpenAI, San Francisco, CA, USA) as a visualization aid. The AI-generated outputs were subsequently reviewed, edited in Adobe Photoshop (Adobe Inc., San Jose, CA, USA), and validated by the authors to ensure scientific accuracy and consistency with the content of this review. The authors assume full responsibility for the final content of all figures.

## 3. RILD: Clinical Presentation and Epidemiology

RILD is a clinically heterogeneous complication of hepatic irradiation. Two principal clinical patterns are recognized: classical RILD and non-classical RILD. Classical RILD typically develops in patients without substantial pre-existing hepatic dysfunction and is characterized by anicteric hepatomegaly, ascites, and, in some cases, an isolated elevation of alkaline phosphatase. It generally develops within several weeks to months after irradiation, most commonly within the first 4 months [11]. In contrast, non-classical RILD occurs predominantly in patients with underlying chronic liver disease, particularly cirrhosis or viral hepatitis, and is characterized by deterioration of liver function, including elevation of serum transaminases and worsening of the Child–Pugh score [11,12].

In patients with cirrhosis, radiation-associated hepatic injury should be distinguished from hepatic decompensation, because radiation may precipitate or accelerate deterioration in a liver with limited baseline functional reserve. In this setting, clinically relevant toxicity may manifest as worsening synthetic dysfunction or other complications of advanced liver disease rather than the characteristic presentation of classical RILD [11,12]. Accordingly, assessment of radiation-related hepatic toxicity should incorporate both the conventional clinical manifestations of RILD and changes in baseline hepatic function.

The risk of RILD is determined by an interaction between patient-related factors, baseline hepatic reserve, tumor characteristics, prior treatment, and radiation dose distribution. Major patient-related risk factors include baseline Child–Pugh class, ALBI (albumin-bilirubin) grade, cirrhosis, viral hepatitis, portal hypertension, previous liver-directed treatment, concomitant systemic therapy, tumor burden, and remaining functional liver volume (Table 1). These factors collectively influence the hepatic reserve available to tolerate additional radiation exposure.

Beyond patient-related factors, dosimetric parameters—including the mean liver dose (MLD) and the volume of liver receiving ≥20 Gy (V20) or ≥30 Gy (V30)—are essential components of modern risk assessment, with MLD < 30 Gy and V30 < 30% generally associated with lower RILD risk. These dose-volume constraints must be interpreted in the context of baseline hepatic reserve, as patients with pre-existing liver dysfunction require more stringent dose limits [16,17].

The epidemiology of RILD reflects the interaction between radiation dose, irradiated liver volume, treatment technique, and hepatic reserve. While dose–response relationships have been established in historical series using earlier RT techniques, these figures cannot be directly extrapolated to contemporary highly conformal approaches. In modern SBRT, the incidence and clinical definition of RILD differ according to baseline liver function and the criteria used to define toxicity. In a cohort of 117 patients with HCC treated with SBRT, RILD occurred in 29 patients (24.7%) during a median follow-up of 22.5 months [13].

Thus, RILD should not be regarded as a complication determined by radiation dose alone. Contemporary risk assessment requires integration of baseline hepatic reserve, underlying liver disease, tumor burden, prior and concomitant therapies, and normal-liver dose–volume parameters. This distinction is particularly important in patients with cirrhosis, in whom radiation-associated injury may manifest primarily as hepatic decompensation rather than classical RILD.

## 4. Radiation Therapy Methods for Malignant Neoplasms of the Abdominal Cavity

Currently, there are many classifications and nosologies of RT methods and types. According to the WHO definition, IR is a type of energy released by atoms in the form of electromagnetic waves or particles [18]. By type of particles, IR is divided into alpha radiation (helium atom nuclei: 2 protons + 2 neutrons), beta radiation (electrons, protons), photons, X- and gamma-wave radiation [19]. These types of radiation are listed in order of increasing penetrating ability. In the treatment of malignant neoplasms (MN), wave types of IR are most commonly used, the energy of which is capable of destroying highly proliferating atypical cells in accordance with the Bergonie–Tribondeau law [20].

In clinical practice, the selection of IR type, dose and irradiation regimen, as well as the method of exposure, is determined based on many factors: tumor size, operability, localization, degree of differentiation, morphology, structure, metastasis, etc. In addition, patient data are taken into account, such as age, gender, lifestyle, risk factors for disease and complication development, concomitant diseases, etc. Based on all the listed data, the physician establishes the goals and objectives of radiation exposure: mono- or complex (in combination with surgical treatment/chemotherapy), adjuvant, neoadjuvant, palliative therapy [21].

Based on the above, it becomes clear that the choice of dose, regimen and type of RT varies widely. For example, for retroperitoneal non-organ sarcomas, conformal RT using IMRT (intensity-modulated RT) in fractionation mode at a total focal dose (TFD) of 45–50 Gy is recommended, which is justified due to the high risk of tumor recurrence and low frequency of metastasis. With this treatment, abdominal organs are “organs at risk” of radiation damage [22]. In addition, external-beam RT can be prescribed for the treatment of chronic pain in colorectal cancer to eliminate disease symptoms [23].

The role of RT in HCC has evolved considerably. According to the NCCN Clinical Practice Guidelines (Version 2.2026), RT is an established treatment option for patients with unresectable disease or those who are medically inoperable. SBRT may be used as an alternative to thermal ablation or embolization when these approaches are contraindicated or unsuccessful, whereas IMRT and proton beam therapy are selected according to tumor number and location, baseline liver function, proximity to radiosensitive organs, and the feasibility of image guidance and respiratory motion management. Contemporary guidelines also provide disease-specific fractionation schedules. For HCC, SBRT is typically delivered at 27.5–60 Gy in 3–5 fractions (BED10 > 72 Gy) when normal liver constraints can be respected. Alternative regimens include hypofractionated RT at 37.5–72 Gy in 10–15 fractions and conventional fractionation at 50–66 Gy in 25–33 fractions. Patients with Child–Pugh B7 cirrhosis generally require dose reduction and stricter normal liver constraints, whereas the safety of RT has not been established for Child–Pugh C cirrhosis [24]. For biliary tract cancers (BTCs), the NCCN Guidelines (Version 1.2026) recommend postoperative RT using conventional fractionation at 45 Gy in 1.8 Gy/fraction to regional lymph nodes and 50–60 Gy in 1.8–2.0 Gy/fraction to the tumor bed depending on margin positivity. For unresectable BTCs, SBRT at 40–60 Gy in 3–5 fractions (BED10 > 100 Gy) is preferred if dose constraints can be met, with alternative hypofractionated regimens of 58–67.5 Gy in 15 fractions or conventional fractionation up to 60 Gy in 30 fractions [25]. For pancreatic adenocarcinoma, the NCCN Guidelines (Version 3.2026) recommend adjuvant RT at 45–50.4 Gy in 1.8–2.0 Gy fractions (25–28 fractions) to the tumor bed and regional lymph nodes. In the neoadjuvant setting for borderline resectable disease, doses of 36 Gy in 2.4 Gy fractions or 45–54 Gy in 1.8–2.0 Gy fractions have been reported. For locally advanced disease, chemoradiation is delivered at 45–56 Gy in 1.8–2.2 Gy fractions, while SBRT may be given as 30–45 Gy in 3 fractions or 25–50 Gy in 5 fractions. Palliative single-fraction SBRT (8 Gy) may be used for celiac axis pain relief [26]. For gastric cancer, postoperative adjuvant RT is delivered at 45–50.4 Gy in 25–28 fractions of 1.8 Gy per day, while external-beam RT may be used for palliation of bleeding and pain [27]. In contrast, gastrointestinal stromal tumors are primarily managed with surgery and tyrosine kinase inhibitors, with radiotherapy reserved for palliative treatment of symptomatic bone metastases or limited progressive disease not amenable to resection or ablation [28].

## 5. Electron Irradiation

One of the promising and poorly studied types of IR is electrons (β-particles), which have relatively low mass. Due to low ionizing ability (compared to α-particles), a stream of electrons is capable of penetrating approximately 2–3 cm into biological tissue [9]. However, in clinical practice, electrons are most often compared with photon irradiation (gamma/X-rays) due to their widespread use in RT (Figure 1).

Unlike photons, electron range in tissue is governed primarily by initial kinetic energy and tissue mass stopping power rather than particle mass per se. The clinically relevant therapeutic range is conventionally defined by the depth of the 90% dose level (R90), approximated as R90 (cm) ≈ E/3.2, and the 80% dose level (R80), approximated as R80 (cm) ≈ E/2.8, where E is the most probable electron energy (MeV) at the phantom surface [29]. However, the penumbra width increases with energy and depth, with a bulging of low-dose regions due to increased electron scattering angles, resulting in dose spread beyond the field opening. Irregular surfaces create hot and cold spots: depressions increase the dose below them while decreasing dose around their periphery, whereas protrusions have the opposite effect. Bone shifts the dose toward the surface due to increased stopping power, while air cavities introduce hot and cold spots due to loss of side-scatter equilibrium [30]. These physical limitations are particularly relevant to hepatic anatomy, given the irregular, curved surface of the liver, its proximity to ribs, lung, and air gaps, and the requirements of intraoperative or highly conformal setups [29].

According to recent dosimetric studies, the optimal energy for standard linear electron accelerators for superficial lesions is in the range of 4–6 MeV; however, for the treatment of deeper-seated tumors (including some liver lesions), energies of 6–20 MeV may be required, as this range provides a therapeutic depth of approximately 1.9–6.3 cm [31,32]. Modified clinical accelerators can achieve energies above 20 MeV (for FLASH irradiation at >40 Gy/s over very short time scales), extending the range of possible applications [31,32]. Recently, studies have appeared devoted to assessing the effectiveness of VHEE irradiation (energy 100–250 MeV); data on its safety are contradictory and scarce. Nevertheless, even the use of electron beams with energy of 100 MeV demonstrated a significant reduction in the negative impact of irradiation on healthy “organs at risk” compared to standard photon therapy methods [33].

Electron arc therapy provides excellent dose distribution for superficial tumors along curved surfaces, such as the chest wall, ribs, and limbs, and is particularly useful when tumor extension along the chest wall would require excessive lung irradiation with tangential photon fields. However, this technique requires specialized collimation at the patient surface to sharpen dose falloff at the arc limits [29].

An important aspect of electron irradiation is the preservation of relative safety for healthy cells with high effectiveness against neoplasms, which was proven in recent large studies devoted to comparing electrons and other types of IR within intraoperative RT (IORT) in FLASH and VHEE (Very High-Energy Electron) modes [34,35].

## 6. Molecular Mechanisms of Radiation Damage

The molecular mechanisms described in this section—including DNA damage, oxidative stress, inflammatory signaling, and miRNA-mediated regulation—are largely generic to low-LET ionizing radiation (photons, gamma rays, and electrons) and are not specific to electron irradiation. Unless explicitly indicated, the cited studies were performed using photon or gamma sources, and their findings should not be automatically attributed to electron beams without appropriate experimental validation. This distinction is important because, although the underlying molecular pathways are similar, the spatial and dosimetric characteristics of electron irradiation (limited penetration, steep dose falloff, and lateral scatter) may influence the manifestation of these mechanisms in a tissue-specific manner, particularly in complex anatomical regions such as the liver.

Numerous studies have shown that IR exposure leads to radiation damage of all biological tissues falling into the irradiation zone with disruption of structure and function of both atypical and healthy cells. Moreover, there are data on the development of the so-called “bystander effect” (or radiation-induced “bystander” effect) [36], the molecular mechanisms of which remain a subject of discussion and are probably associated with activation of cell proliferation or death, toxic effects of free radicals, induction of gene expression of mitogen-activated protein kinase (MAPKs) signaling pathways, nuclear factor kappa B (NF-κB), nitric oxide synthase (iNOS) and cyclooxygenase-2 (COX-2) [37]. These changes lead to the death of adjacent healthy cells not directly falling into the irradiation zone, which is associated with an increase in the irradiated zone and its spread around the focus of radiation exposure.

Cytotoxic effects of various types of IR are quite uniform and occur according to general principles in most organs. Thus, a stream of charged particles (during beta irradiation) or waves (during gamma irradiation) launches direct and indirect pathways of radiation damage.

### 6.1. Direct Pathway

The direct pathway is due to damage to the genetic apparatus and macromolecules in cells directly by the energy of a charged particle. A stream of electrons “knocks out” particles from DNA chain atoms, leading to changes in the charge of their individual sections. This is accompanied by the formation of breaks and cross-links in the DNA structure, chromosomal aberrations. Thus, single-strand DNA breaks/cross-links are considered less dangerous due to the possibility of their repair from the second chain matrix [38]. Nevertheless, in case of non-reparable damage with the participation of transforming growth factor-α (TGF-α) and tumor necrosis factor-α (TNF-α), activation of MAPK/Jnk/p38 cascades occurs, the modulation of which is responsible for triggering cell death through apoptosis, as well as for the development of the previously described “bystander effect” [39].

In turn, double-strand DNA breaks/cross-links are most often fatal for the cell, as their repair is practically impossible [40]. Their appearance is associated with activation of gamma-H2AX loci and recruitment of DNA damage marker proteins (KU70, KU80). Gamma-H2AX phosphorylation leads to induction of MAPK/Jnk/p38, which triggers cell death through apoptosis [41].

In addition, in some cases, accumulation of whole clusters and complexes of DNA damage (cluster/complex DNA damage, CDD) occurs, which significantly slows down repair and leads to accumulation of a large number of non-reparable mutations, increasing genome instability [42].

Furthermore, in isolated molecular-genetic studies, it was found that some micro-RNAs (miR) are also direct participants in radiation damage. Thus, miR-34a is a well-established mediator of p53-dependent apoptosis and G1 cell cycle arrest in response to DNA damage [43]. In the context of HCC, Zhao et al. demonstrated that miR-34a-5p enhances radiosensitivity by targeting c-MYC and repressing CHK1/CHK2-mediated DNA damage response, thereby counteracting cancer stem cell-like properties and overcoming radioresistance [44]. miR-34a increases p53 protein levels and stability, creating a positive feedback loop that induces p53-mediated apoptosis, cell cycle arrest, and senescence in radiation-induced liver injury mouse models [45]. In another study, it was found that miR-21 and miR-34c are responsible for inducing the “bystander effect” by increasing the expression of TGF-β1 and NO factors, while miR-495 helps limit the “bystander effect” by modulating the Sp1/eNOS signaling pathway (decreased NO) [46,47,48]. Consistent with this, miR-495 has been shown to alleviate radiation-induced cell injury in mice by indirectly downregulating eNOS and NO production via targeting Sp1, thereby inhibiting NO and its downstream product TGF-β1 [49].

In contrast to these pro-inflammatory miRNAs, miR-146a-5p has been identified as a protective factor against RILD. Mechanistically, it suppresses irradiation-induced HSC activation and hepatocyte apoptosis via inhibition of the TLR4 signaling pathway, leading to reduced production of pro-inflammatory cytokines [50]. Additionally, miR-146a-5p negatively regulates the PTPRA-SRC signaling axis, thereby inhibiting the expression of fibrosis-related markers such as α-SMA and collagen 1 in irradiated HSCs [51].

Beyond miRNA-mediated mechanisms, recent evidence highlights the involvement of long non-coding RNAs (lncRNAs) in radiation damage. Notably, the lncRNA NEAT1 acts as a molecular sponge for miR-147, thereby regulating PDPK1 expression and AKT activation; treatment with the radioprotective agent troxerutin upregulated NEAT1, which sequestered miR-147 and restored PDPK1, leading to reduced apoptosis [52]. A comprehensive review further summarizes the involvement of numerous miRNAs in DNA damage response, cell cycle regulation, and apoptosis, highlighting radiation-associated miRNAs in HCC such as miR-146a-5p (targeting RPA3), miR-101-3p (via NEAT1_2 sponging), miR-203, miR-106a, miR-621, and miR-26b [53].

### 6.2. Indirect Pathway

The indirect pathway of tissue radiation damage is due to the ionizing ability of alpha-, beta- and gamma radiation. Thus, ionization of atoms during radiolysis of intracellular water leads to their dissociation and generation of free radicals–reactive oxygen species (ROS) and reactive nitrogen species (RNS) [54]. Accumulation of these substrates becomes the cause of oxidative stress development, predominance of oxidants in tissue, and disruption of oxidant–antioxidant homeostasis (endogenous redox system). In addition, ROS are capable of directly damaging DNA and genes responsible for the synthesis of antioxidant defense enzymes, which also exacerbates the degree of oxidative damage [55].

Biochemically, ROS are most often products of water molecule radiolysis, that is, radicals containing oxygen, with a deficit of one to three electrons, which significantly increases their reactivity [56]. Under oxidative stress caused by IR, hydrogen peroxide (H_2_O_2_), singlet oxygen (^1^O_2_), superoxide anion radical (O_2_^•−^) and hydroxyl radical (•OH) usually predominate [57]. The latter is considered one of the most dangerous, as it is capable of oxidizing a large number of intracellular macromolecules, such as proteins, lipids, carbohydrates, etc. [58].

In turn, as a result of lipid molecule peroxidation by oxygen free radicals, lipid hydroperoxides (LOOH) are formed, which are also capable of interacting with other macromolecules and enhancing their degradation within oxidative stress [59]. The end products of this process are considered to be two of the most studied compounds, the levels of which are used to assess the severity of lipid peroxidation and oxidative stress–malondialdehyde (MDA) and trans-4-hydroxy-2-nonenal (4-HNE) [60].

In addition, the effect of oxidative stress on protein macromolecules is well studied, which not only change their conformation with disruption of biological functions, but also interact with free radicals with the formation of carbonyl protein derivatives [61]. Furthermore, as a result of free radical exposure during IR, DNA structure disruption is possible, both nuclear and mitochondrial, which leads to mutations and even carcinogenesis. Similar mechanisms of oxidative stress effects on cells are described in some oncological, cardiovascular, endocrine, neurodegenerative, allergic and other diseases [62,63,64,65].

The endogenous antioxidant system is an adaptive mechanism of organism protection against free radicals generated in response to IR exposure. Its key effectors are endogenous low-molecular-weight antioxidants (glutathione, ascorbate, coenzyme Q10, alpha-lipoic acid, tocopherol, etc.) and antioxidant enzymes (superoxide dismutases (SODs), glutathione reductase (GR), glutathione peroxidases (GPxs) and catalases (CAT)) [60,66]. Exogenous antioxidants also participate in protection against the toxic effects of free radicals: carotenoids, vitamins A, C, E, polyphenols, selenium, etc. [67]. However, IR exposure is usually associated with rapid ROS synthesis and leads to mitochondrial dysfunction combined with dysregulation of iNOS, lipoxygenases, cyclooxygenases and NADPH oxidases activity [68]. Thus, the antioxidant system is quickly depleted and an imbalance of oxidants and antioxidants is formed with a pronounced predominance of the former over the latter. Continued free radical generation during redox system dysfunction leads to further damage to irradiated (as a result of indirect radiation damage mechanisms) and healthy (“bystander effect”) cells of the organ. In addition, the synthesis of numerous cytokines combined with the formation of a large number of cell destruction products activates the migration of immune cells to the irradiation zone, which also express a large number of cytotoxic factors, damaging healthy cells in the process. The molecular mechanisms of this damage pathway will be described below.

## 7. Effects of IR on the Liver

The following section describes established pathological features of RILD in humans where available. Mechanistic pathways demonstrated exclusively in cell lines or animal models are explicitly identified as such. This distinction is critical, as not all experimental findings translate directly to clinical RILD.

RILD is a complex pathological condition arising as a result of IR exposure to liver tissues, leading to various molecular and cellular changes (Figure 2). The main pathogenetic mechanisms of RILD development include DNA damage (direct pathway of radiation damage), oxidative stress (indirect pathway of radiation damage), inflammation (acute or chronic) and fibrosis (in the long term).

### 7.1. Oxidative Stress and Mitochondrial Damage

In human studies, oxidative stress markers are elevated in patients with RILD [68]. In animal models, mitochondrial damage leads to disruption of electron transport chain functions and enhanced ROS formation, which causes apoptosis and premature death of liver cells. ROS accumulation enhances damage to cell membranes, proteins and DNA, which initiates mitochondria-mediated necrosis and the mitochondrial (internal) pathway of apoptosis [69,70,71]. In biochemical and cell-based studies, the intrinsic apoptotic pathway has been characterized in detail: following its release from mitochondria, cytochrome c binds to apoptotic protease-activating factor-1 (Apaf-1), promoting apoptosome formation and activation of caspase-9. Activated caspase-9 subsequently cleaves and activates the effector caspases-3 and -7, which execute the apoptotic program and ultimately result in cell death [72].

### 7.2. DNA Damage and Cellular Senescence

Radiation causes direct DNA damage, generating single-strand and double-strand breaks, which activate DNA repair mechanisms and the DNA Damage Response (DDR) system [3,55]. In human RILD, these processes contribute to hepatocyte loss and liver dysfunction. In human and mouse cell lines, as well as in vitro biochemical studies, key proteins ATM and ATR recognize damage and activate signaling cascades for cell cycle arrest and initiation of DNA repair. If damage is too extensive and cannot be corrected, these signaling pathways trigger apoptosis through p53 activation and pro-apoptotic proteins of the Bcl-2 family, such as Bax and Bak, which promote cytochrome c release from mitochondria and initiation of the internal apoptotic cascade through caspases 9 and 3, leading to degradation of cellular components and hepatocyte death [73].

In addition to classical apoptosis pathways, cell and animal model studies have demonstrated that radiation can cause ferroptosis—a special form of cell death associated with lipid oxidation and iron metabolism disruption. IR stimulates the accumulation of labile iron, which leads to the formation of toxic lipid oxidation products and subsequent cell death [74].

Significant DNA damage that does not trigger apoptosis can lead to cellular senescence—a stable state of proliferation arrest that acts as a protective mechanism against tumorigenesis. However, the accumulation of senescent cells in the liver has been associated with chronic inflammation and fibrosis in experimental models. For instance, in a rat model of CCl_4_-induced liver fibrosis, senescence of activated HSCs via the STAT3-p53 pathway was shown to limit fibrosis progression [75]. In cell and animal models, senescent cells have been shown to secrete pro-inflammatory factors such as interleukin-6 (IL-6) and interleukin-1β (IL-1β), as well as growth factors, forming the so-called senescence-associated secretory phenotype (SASP) [76,77]. Whether these senescence-associated mechanisms contribute to radiation-induced liver damage in humans remains to be elucidated.

### 7.3. Inflammation

Practically all types of IR act on both hepatocytes and resident macrophages–Kupffer cells, primarily stimulating secretion of cytokines such as TNF-α and TGF-β [3,78]. In irradiated rat and mouse models, Kupffer cells have been shown to release TNF-α in a dose-dependent manner, which promotes hepatocyte apoptosis when co-cultured with irradiated hepatocytes. Activated Kupffer cells secrete TGF-β, the major profibrogenic cytokine, which drives the transdifferentiation of quiescent HSCs into myofibroblasts, the principal collagen-producing cells in the liver [3,78]. In addition to direct DNA damage responses, DDR activation causes transcription of pro-inflammatory genes through the NF-κB signaling pathway, leading to production of cytokines such as TNF-α, IL-1β and IL-6. The NF-κB signaling pathway is a central regulator of the inflammatory response during radiation damage [78,79]. In experimental models of acute radiation-induced liver injury, ROS and DDR also activate the JAK/STAT signaling pathway, leading to expression of genes responsible for the production of pro-inflammatory cytokines and modulation of the inflammatory response [80]. In an in vivo rat model, over-activation of JAK1/STAT3 was associated with hepatic inflammation and ferroptosis following gamma-irradiation [80]. The p38 MAPK pathway is another key mediator of the cellular response to ionizing radiation, activated through ROS generation and involved in the regulation of inflammatory and fibrotic responses in various experimental models [81]. Inhibition of p38 MAPK has been shown to reduce radiation-induced fibrosis in healthy tissues, suggesting its potential role in the development of late radiation sequelae [81]. However, the precise contribution of p38 MAPK to chronic inflammation during fractionated irradiation remains to be fully elucidated.

In addition to the well-established clinical manifestations of RILD in humans, the SASP pattern, a key feature of radiation-induced cellular senescence observed in animal models, contributes to the secretion of pro-inflammatory and profibrotic mediators (IL-6, IL-1α/β, TNF-α, TGF-β), which promote immune cell migration, chronic inflammation, and activation of fibroblasts and HCS [45]. For example, Zhu et al. demonstrated that a single dose of 25 Gy radiation induced hepatocyte senescence in rats, as evidenced by upregulation of SA-β-gal, increased cell size, elevated p16 and p21 expression, and activation of SASPs including IL-6 and IL-1α [82].

In vitro and in vivo studies have demonstrated that liver endothelial cells are also radiosensitive and play an important role in the development of the inflammatory response. In human umbilical vein endothelial cells (HUVECs), irradiation at doses of 2–4 Gy induces senescence and upregulates adhesion molecules such as ICAM-1 and VCAM-1, promoting the recruitment of inflammatory cells, including neutrophils and monocytes [83]. These findings have been corroborated in vivo in rodent models, where whole-body irradiation of rats (10 Gy) increased the expression of pro-inflammatory cytokines, and endothelial dysfunction was observed in mice following cardiac irradiation [83].

Furthermore, some authors report enhanced inflammation and fibrosis in surrounding liver tissues with the development of the “bystander effect,” the pathogenesis of which was described earlier [36].

In combination, the listed molecular mechanisms lead to structural changes in the liver as a result of mixed (apoptosis, ferroptosis, mitochondria-mediated necrosis) hepatocyte death, combined with endothelial dysfunction, inflammation (initially acute, then chronic), fibrosis and progressive liver failure, which is considered a RILD pattern (Table 2).

## 8. Radiation-Induced Liver Fibrosis

RILD develops as a consequence of radiation therapy and is characterized by significant damage to liver tissue, leading to progressive fibrosis. Liver fibrosis is a complex process involving activation of HSCs, chronic inflammation and extracellular matrix (ECM) remodeling. These changes are based on molecular mechanisms associated with oxidative stress, activation of signaling pathways and production of pro-inflammatory cytokines [84,85]. SASP-derived mediators further amplify fibrogenesis by activating HSCs, macrophages, and endothelial cells in a self-sustaining cycle of inflammation and ECM remodeling.

Evidence specific to electron-beam irradiation of the liver remains limited and is drawn largely from intraoperative electron radiotherapy (IOERT) settings, where the shallow penetration depth of electrons concentrates dose in the resection bed and adjacent parenchyma rather than producing the whole-organ dose distribution typical of external-beam photon series; oxidative stress has been proposed as a key contributor to liver injury in this setting [86]. In this model, male Wistar rats underwent fractionated local electron irradiation of the upper abdomen (6 × 5 Gy at 24 h intervals, 30 Gy total, 10 MeV, NOVAC-11 linear accelerator), with animals assessed on days 7, 30, 60, and 90; an acute biochemical and inflammatory response (elevated ALT, AST, ALP, bilirubin, IL-1β, IL-6, and TNF-α) was evident as early as day 7, whereas histologically established fibrotic remodeling of the liver parenchyma emerged only by days 60–90 [86]. This temporal pattern illustrates the distinction, also seen clinically, between an early reversible inflammatory response and a later, structurally fixed fibrotic lesion. The latency period between irradiation and clinically apparent fibrosis can range from several months to years, with late fibrosis often manifesting 6–12 months or more after treatment, depending on dose and irradiated volume [3].

The molecular events downstream of HSC activation are largely conserved across etiologies of liver injury (toxic, viral, radiation-induced) and have been characterized predominantly in non-radiation models of hepatic fibrosis—chiefly CCl4-induced injury in rats and mice, and in vitro HSC cultures [87,88,89,90,91,92,93,94,95,96,97,98,99]; their applicability to RILD specifically is inferred rather than directly demonstrated.

At the tissue level, HSCs are central to the fibrotic response [87]. Quiescent, vitamin A-storing HSCs transdifferentiate into myofibroblast-like cells under the influence of ROS and pro-inflammatory mediators, producing type I/III collagen, fibronectin, and laminin; TGF-β, PDGF, and Ang II are the principal activators [88]. In a rat model of radiation-induced liver fibrosis, targeted inhibition of TGF-β signaling using an adenoviral vector expressing a soluble TGF-β type II receptor–Fc fusion protein (AdTβRIIFc) significantly reduced hepatic fibrosis, decreased oxidative stress damage, and suppressed HSC activation, indicating that TGF-β is a critical driver of fibrogenesis in the irradiated liver [100]. TGF-β acts through Smad2/3–Smad4 nuclear signaling to drive fibrogenic gene expression, alongside non-Smad pathways (MAPK, PI3K/AKT, Rho/ROCK) that sustain HSC proliferation, migration, and survival [89,97]. SASP-derived mediators and reciprocal crosstalk between HSCs, Kupffer cells, sinusoidal endothelial cells, and senescent hepatocytes—via cytokines such as IL-6 and MCP-1—establish a self-sustaining cycle of inflammation and extracellular matrix (ECM) remodeling [90,91,92,93]. Progressive fibrosis further reflects an imbalance between matrix metalloproteinases (MMPs) and their inhibitors (TIMPs), which TGF-β shifts toward reduced ECM degradation [90,94], while the Smad7-mediated negative feedback that normally limits Smad2/3 activation is itself suppressed in chronic liver injury, permitting unchecked fibrogenesis [95,96,98,99].

Recent mechanistic insights have expanded the understanding of radiation-induced liver fibrosis beyond classical TGF-β/Smad signaling. Liu et al. proposed that ionizing radiation-induced extracellular matrix stiffening activates the mechanosensitive ion channel Piezo1, which transduces mechanical stimuli into biochemical signals through the HIF-1α/TGF-β pathway, driving the differentiation of FAP+ cancer-associated fibroblasts (CAFs) and pathological ECM remodeling [101]. This Piezo1-mediated mechanotransduction also promotes macrophage polarization toward pro-fibrotic phenotypes, creating a self-reinforcing cycle of fibrosis and matrix stiffening [101]. Although primarily a mechanistic hypothesis based on emerging experimental evidence, this Piezo1-FAP+ CAF axis offers a promising novel target for mitigating radiation-induced liver fibrosis.

It should be noted that most of the mechanistic evidence above derives from rodent models of whole- or partial-liver irradiation assessed by histological collagen quantification, which captures the fibrotic process at the tissue level but does not, by itself, establish a direct correlate with clinical RILD or hepatic decompensation in patients; the relationship between histologically demonstrable fibrosis and clinically meaningful liver dysfunction remains incompletely defined and warrants cautious extrapolation.

## 9. Radioprotective Properties of Amifostine

Radioprotectors are substances used to protect the body from damage caused by IR. Their development and use are aimed at reducing radiation damage in areas such as RT, space medicine, and emergency situations associated with radiation exposure [102].

In contrast to radiation mitigators, which are administered after irradiation to reduce its negative effects, radioprotectors are administered prior to irradiation to provide a high protective effect, manifested by reduced damage to radiosensitive tissues and accelerated post-radiation recovery [103]. The main goal of radioprotectors is to minimize the harmful effects of IR on living cells and tissues [102]. Radioprotectors can be divided into two main groups based on the duration of their protective effect: short-acting and long-acting. Short-acting radioprotectors are effective during pulsed or short-term radiation exposure. Their protective effect manifests within the first minutes after administration and lasts from 30 min to several hours. In this group, sulfur-containing compounds (e.g., cysteine and aminothiols), antioxidants (such as ascorbic acid and tocopherol), and substances causing cell hypoxia are particularly distinguished. Long-acting radioprotectors provide protection during prolonged irradiation. Their action can last from several hours to several days. This category includes substances with anabolic properties, polysaccharides, and polynucleotides [103].

Amifostine (Ethyol^®^, WR-2721) is the most extensively studied synthetic radioprotector and remains the only FDA-approved agent for radiation protection [104]. Following intravenous administration, amifostine is rapidly dephosphorylated by alkaline phosphatase to its active metabolite WR-1065, which is responsible for its radioprotective effects [105]. Its mechanisms of action include scavenging of reactive oxygen species, enhancement of DNA repair, and induction of transient cellular hypoxia [106,107,108,109,110,111].

According to the FDA-approved prescribing information, amifostine has two specific indications:

(1) Reduction of cumulative renal toxicity associated with repeated administration of cisplatin in patients with advanced ovarian cancer [112]. The recommended dose is 910 mg/m^2^ administered as a 15-min intravenous infusion, starting 30 min prior to chemotherapy [112].
(2) Reduction of the incidence of moderate to severe xerostomia in patients undergoing postoperative radiation treatment for head and neck cancer, where the radiation port includes a substantial portion of the parotid glands [112]. The recommended dose is 200 mg/m^2^ administered as a 3-min intravenous infusion, starting 15–30 min prior to standard fraction radiation therapy (1.8–2.0 Gy) [112].

The FDA label explicitly warns against the use of amifostine in settings where chemotherapy can produce a significant survival benefit or cure, or in patients receiving definitive radiotherapy, except in the context of a clinical study [112].

The main molecular effects of this drug are as follows:

1. Amifostine acts as a “trap” for hydroxyl radicals and other ROS, which reduces the degree of oxidative stress and, consequently, the amount of DNA, lipid and protein damage [106,107].
2. Amifostine can inhibit radiation-induced caspase activation, which triggers apoptosis in response to critical DNA damage [108].
3. Amifostine stimulates the expression of endogenous antioxidant enzymes such as SOD and GPx. These enzymes play an important role in ROS detoxification and cell protection from oxidative stress, which additionally enhances the radioprotective potential of the drug [109,110].
4. One of the important features of amifostine is its selective accumulation in normal tissues. This is associated with higher alkaline phosphatase activity in normal cells compared to tumor cells, leading to preferential conversion of amifostine to its active form specifically in healthy tissues. This ensures protection of normal tissues with minimal impact on the tumor [111].

Amifostine has long been considered a well-studied radioprotective drug with proven effectiveness. Nevertheless, despite its advantages, the use of amifostine is associated with significant clinical limitations, including frequent adverse effects such as nausea, vomiting, and hypotension, which occur in the majority of patients at therapeutic doses [112]; the potential to interfere with the antitumor efficacy of chemotherapy or radiotherapy in certain settings [112]; the requirement for intravenous administration, which complicates outpatient use and necessitates close medical supervision [104]; a narrow therapeutic window; and high cost, further restricting its routine clinical application [104].

## 10. Ascorbic Acid as a Radioprotective Agent

### 10.1. Rationale and Biological Basis

Given the central role of oxidative stress in RILD pathogenesis, the potential of antioxidant vitamins as radioprotective agents has been extensively investigated. A systematic review by Lledó et al. evaluated ex vivo, in vitro, and in vivo evidence on vitamin-mediated radioprotection and identified vitamins A, C, D, and E as the principal vitamins for which radioprotective effects have been reported [113]. The reported effects include attenuation of oxidative damage, reduction of radiation-induced DNA damage, inhibition of lipid peroxidation, and modulation of cellular stress and inflammatory responses. However, the evidence differs substantially among vitamins and experimental settings, and most studies remain preclinical.

Vitamin E has demonstrated radioprotective effects in experimental models of gastrointestinal and hematopoietic radiation injury and has also been evaluated clinically for prevention of radiation-associated salivary gland injury following radioiodine therapy [114,115,116]. Vitamin D has been reported to attenuate irradiation-induced endothelial cell senescence and apoptosis through modulation of the MAPK/Sirt1 pathway [117]. Vitamin A has also been investigated as a radioprotective agent, with protective effects reported in combination with magnesium sulfate in an experimental mouse model, including reduction of radiation-induced micronuclei and modulation of NOX4 expression [118]. These findings support the biological plausibility of vitamin-mediated radioprotection but do not establish equivalent efficacy or clinical applicability across the different vitamins.

Among these candidates, we selected ascorbic acid (vitamin C) as the primary focus of this section because of the relatively broad experimental evidence supporting its antioxidant and radioprotective properties, as well as its direct relevance to several mechanisms implicated in radiation-induced tissue injury. Vitamin C has been shown to reduce radiation-induced DNA and chromosomal damage in human lymphocytes and animal models [119,120,121] and to attenuate radiation-associated oxidative and molecular damage [122,123,124]. Protective and mitigating effects of vitamin C have also been investigated in experimental models of radioiodine-induced toxicity [125].

### 10.2. Experimental Evidence

Experimental evidence for the radioprotective effects of ascorbic acid has been obtained in several models and under substantially different irradiation conditions. In the mouse model of whole-body gamma irradiation, high-dose ascorbic acid administered intraperitoneally at 3 g/kg immediately after exposure to 7–8 Gy increased survival, reduced radiation-induced bone-marrow apoptosis, and promoted hematopoietic recovery. Administration of 3 g/kg remained effective when given at 1, 6, 12, or 24 h after 7.5 Gy irradiation, whereas treatment initiated more than 36 h after irradiation was ineffective. A divided regimen of 1.5 g/kg immediately and 1.5 g/kg at 24 h was also effective. In contrast, doses of ≥4 g/kg administered after irradiation were harmful, demonstrating a narrow experimental dose range [126]. More directly relevant to hepatic radiation injury, a recent study evaluated vitamin C in adult male NMRI mice without tumors exposed to whole-body 60Co gamma-radiation at 2 or 4 Gy. Vitamin C was administered by oral gavage at 50, 100, or 200 mg/kg twice daily for 3 days before irradiation, with irradiation performed approximately 2–3 h after the final administration. Liver tissue was examined 48 h after irradiation. Vitamin C, particularly at 100–200 mg/kg, was associated with changes in hepatic lipid peroxidation and glutathione parameters consistent with attenuation of radiation-induced oxidative stress. However, this study assessed biochemical markers of acute hepatic oxidative injury rather than clinically defined RILD or hepatic decompensation [127]. Evidence also exists for an ascorbic-acid derivative rather than native ascorbic acid. Ascorbic acid monoglucoside (AsAG) was administered orally to mice before 4 Gy whole-body gamma-irradiation and reduced radiation-induced lipid peroxidation in liver tissue. In vitro, AsAG also reduced lipid peroxidation in mouse liver tissue exposed to 25 Gy γ-radiation and protected plasmid DNA from radiation-induced strand breaks. These findings support a potential antioxidant mechanism but cannot be directly extrapolated to clinical use of intravenous or oral ascorbic acid, because AsAG is a chemically distinct compound and the experiments were performed in non-tumor models [128].

### 10.3. Potential Molecular Mechanisms

Ascorbic acid is a water-soluble vitamin with multiple physiological functions, including participation in collagen biosynthesis, immune-cell function, iron metabolism, and cellular antioxidant defense. In its ascorbate form, vitamin C acts as an endogenous water-soluble antioxidant and can participate in the protection of cellular components from oxidative damage [67,129]. Its potential relevance to radiation-induced tissue injury can therefore be considered in the context of several complementary biological mechanisms.

1. Antioxidant Activity

Ascorbate can directly participate in redox reactions and scavenge reactive oxygen species generated during oxidative stress. By limiting the accumulation of reactive species, vitamin C may reduce oxidative damage to DNA, lipids, and proteins following ionizing radiation exposure [67,129]. This mechanism is particularly relevant to RILD because radiation-induced ROS generation contributes to endothelial dysfunction, inflammatory signaling, and subsequent tissue injury.

2. Participation in collagen synthesis and extracellular matrix homeostasis

Ascorbic acid is an essential cofactor for prolyl and lysyl hydroxylases involved in collagen biosynthesis. Through this function, vitamin C contributes to normal collagen maturation and extracellular matrix maintenance [130]. Because radiation-induced tissue remodeling and fibrosis involve alterations in extracellular matrix turnover, adequate vitamin C availability may be biologically relevant to tissue repair. However, direct evidence that vitamin C supplementation prevents radiation-induced hepatic fibrosis remains limited, and the role of vitamin C in collagen metabolism should therefore not be interpreted as evidence of an established antifibrotic effect in RILD.

3. Modulation of immune and inflammatory responses

Vitamin C is involved in multiple aspects of leukocyte function and immune regulation [67,129]. Through its effects on cellular redox balance and immune-cell function, vitamin C may influence inflammatory responses following tissue injury. Nevertheless, evidence specifically demonstrating that vitamin C supplementation attenuates radiation-induced hepatic inflammation remains limited, and this mechanism should therefore be considered biologically plausible rather than clinically established.

4. Interaction with other antioxidant systems

Vitamin C may also interact with other antioxidant systems, including vitamin E, potentially contributing to the maintenance of cellular redox balance and antioxidant capacity [131,132,133]. Experimental and biochemical studies have demonstrated interactions between vitamins C and E during free-radical scavenging, including mechanisms by which vitamin C can contribute to maintenance of the antioxidant state of vitamin E [131,132]. However, available evidence does not establish a consistent synergistic radioprotective effect of combined antioxidant supplementation, and the outcome may depend on the specific compounds, concentrations, administration schedules, and experimental conditions.

In addition to its antioxidant effects, experimental studies have investigated the potential of vitamin C to influence radiation-induced DNA damage through mechanisms involving transition metals. In particular, Cai et al. demonstrated that the effects of vitamin C on radiation-induced DNA damage may depend on the presence or absence of copper, highlighting the context-dependent nature of its redox activity [10]. Accordingly, vitamin C should not be regarded as uniformly protective under all biological conditions. Its antioxidant and potential pro-oxidant effects depend on concentration, redox environment, metal availability, and cellular context [10,134].

### 10.4. Limitations and Future Directions

From a practical perspective, vitamin C is an attractive candidate for further investigation because of its wide availability, relatively low cost, established physiological functions, and potential for combination with other antioxidant agents. These characteristics may be particularly relevant when considering supportive strategies for normal-tissue protection. However, practical accessibility should not be interpreted as evidence of clinical efficacy in patients receiving liver-directed RT.

Experimental studies have predominantly evaluated vitamin C administration before or around the time of radiation exposure, whereas evidence concerning post-radiation administration remains more limited and heterogeneous [135]. Consequently, the potential importance of administration timing should be considered when interpreting experimental findings. The optimal dose, concentration, route, formulation, and timing of vitamin C administration for radioprotection remain to be established, particularly in the context of external-beam RT.

Importantly, the available evidence is heterogeneous with respect to radiation quality, radiation dose, experimental model, vitamin concentration, route and timing of administration, and whether normal tissues or tumors were present during irradiation. Much of the evidence supporting vitamin C as a radioprotective agent derives from in vitro or animal studies, while clinical evidence remains limited and has largely been obtained in radiation-exposure settings other than liver-directed external-beam RT [125]. Therefore, the available evidence does not currently establish vitamin C as a clinically validated radioprotective treatment for RILD.

Our focus on ascorbic acid therefore reflects its mechanistic relevance to oxidative radiation injury and the breadth of experimental evidence available, rather than evidence demonstrating superior radioprotective efficacy compared with vitamins A, D, or E. Further preclinical and clinical studies are required to determine whether vitamin C can provide meaningful protection of normal hepatic tissue without compromising tumor control and to establish appropriate dose, formulation, route, and timing of administration.

## 11. Conclusions

Optimization of RT and preservation of healthy hepatic tissue remain important goals in the treatment of hepatobiliary malignancies. Electron irradiation may provide dosimetric advantages in selected clinical situations because of its finite range and distal dose fall-off; however, its applicability to liver targets is highly dependent on beam energy, target depth and geometry, tissue heterogeneity, field size, surface irregularity, and motion. Current evidence is insufficient to establish clinical superiority of electron therapy over contemporary photon- or proton-based approaches, and further comparative dosimetric and clinical studies are required.

Radiation-induced hepatic injury involves oxidative stress, DNA damage, inflammation, endothelial injury, and fibrosis, but these mechanisms are not established as electron-specific. Radioprotection therefore remains an important complementary strategy. Although amifostine has established clinical indications for cisplatin-associated nephrotoxicity and radiation-induced xerostomia, its use for hepatic radioprotection remains unsupported. Ascorbic acid is a promising experimental candidate because of its antioxidant and redox-regulating properties; however, available evidence is predominantly preclinical and heterogeneous with respect to radiation quality, dose, concentration, route, timing, and experimental model. Its efficacy and optimal administration strategy for hepatic radioprotection remain to be established.

Future research should focus on clinically relevant comparison of electron and photon irradiation, direct assessment of electron-associated hepatic injury, and controlled evaluation of radioprotective strategies such as ascorbic acid, with particular attention to reducing hepatic toxicity without compromising tumor control.:

## Figures and Tables

**Table 1:** Patient-Related Risk Factors for Radiation-Induced Liver Disease (RILD).

Risk Factor	Clinical Significance	Key Evidence/Comments	Refs
Child–Pugh class/score	Reflects baseline hepatic functional reserve and strongly influences susceptibility to radiation-associated liver dysfunction.	In a cohort of 117 patients with HCC treated with SBRT, RILD occurred in 29 patients (24.7%). Pretreatment Child–Pugh score was significantly associated with RILD on univariate analysis (*p* < 0.001) and remained the only significant predictor in multivariable analysis. RILD incidence increased markedly above a Child–Pugh score of 6, while recovery from RILD decreased significantly above a score of 8.	[13]
ALBI grade	Provides an objective assessment of baseline hepatic functional reserve and may improve prediction of post-SBRT hepatotoxicity.	In a retrospective cohort of 121 evaluable HCC patients treated with SBRT, hepatotoxicity occurred in 5%. Baseline ALBI score was an independent predictor of hepatotoxicity and showed greater predictive accuracy than CTP (AUC 0.77). The optimal cutoff was −2.47, with 85.7% sensitivity and 71.1% specificity. Multivariable analysis also identified PTV as a significant factor.	[14]
Cirrhosis	Cirrhosis reduces hepatic functional reserve and shifts the clinical manifestation of radiation injury toward ncRILD and hepatic decompensation.	ncRILD occurs predominantly in patients with underlying chronic liver disease, particularly cirrhosis. In these patients, radiation may impair hepatocellular regeneration and precipitate irreversible hepatic dysfunction.	[11,12]
Viral hepatitis	Chronic viral liver disease may reduce hepatic reserve and increase susceptibility to radiation-associated dysfunction.	Patients with chronic viral hepatitis are particularly represented among ncRILD cases; HBV carriers have been reported to be more vulnerable to RILD than non-carriers. HBV status should therefore be considered when assessing hepatic reserve and treatment tolerance.	[11,12]
Portal hypertension	Indicates advanced chronic liver disease and limited hepatic reserve; clinically important because radiation-associated injury may present as or precipitate hepatic decompensation.	Portal hypertension is a marker of advanced hepatic dysfunction and reduced functional reserve. In patients with cirrhosis, its presence should be considered together with baseline liver function when estimating tolerance to hepatic irradiation.	[11]
Prior liver-directed therapy	Previous local treatment may reduce the volume of functioning liver available for subsequent irradiation and may contribute to cumulative hepatic injury.	Previous liver surgery was evaluated as a potential risk factor in contemporary ncRILD analyses; however, its association was not independent after multivariable adjustment. Previous TACE and other liver-directed treatments should therefore be considered as modifiers of residual hepatic reserve rather than established independent predictors.	[12]
Systemic therapy	Prior or concomitant systemic treatment may contribute to cumulative hepatic toxicity and modify the toxicity profile of RT.	Contemporary ncRILD cohorts include patients receiving immunotherapy and targeted therapy; however, systemic treatment was not an independent predictor of ncRILD in multivariable analysis in the available cohort. Its contribution therefore remains context-dependent.	[5,12]
Tumor burden	Larger or multifocal tumors may reduce the amount of liver that can be spared and may increase the risk of radiation-associated hepatic toxicity.	In an IMRT cohort of 177 patients with HCC, tumor number ≥ 2 was an independent risk factor for ncRILD, whereas larger tumor volume and diameter were associated with ncRILD in univariate analysis. In an SBRT cohort, PTV was independently associated with post-treatment hepatotoxicity together with baseline ALBI score. In 114 patients with Child–Pugh A cirrhosis treated with hypofractionated conformal RT, GTV and V20 were independent predictors of RILD in multivariable analysis.	[12,14,15]
Remaining normal/functional liver volume	The amount of liver spared from irradiation is a major determinant of hepatic tolerance, particularly in patients with limited baseline reserve.	In the 117-patient SBRT cohort, normal liver volume was associated with RILD on univariate analysis, although it was not an independent predictor after adjustment for Child–Pugh score. In a contemporary 177-patient IMRT cohort, normal liver volume < 700 mL was independently associated with ncRILD (OR 3.054, 95% CI 1.114–8.375).	[12,13]

Note: ALBI—albumin-bilirubin; CTP—Child-Turcotte-Pugh; HCC—hepatocellular carcinoma; ncRILD—non-classical RILD; PTV—planning target volume; RT—radiotherapy; RILD—radiation-induced liver disease; SBRT—stereotactic body radiation therapy; TACE—transarterial chemoembolization.

**Table 2:** Pathogenetic Mechanisms of Radiation-Induced Liver Disease (RILD).

Mechanism Category	Trigger/Key Events	Molecular Pathways	Cellular Effectors	Consequences	Refs
DNA Damage (Direct)	Single- and double-strand breaks; DDR activation	ATM/ATR → p53 → Bax/Bak → cytochrome c release	Hepatocytes	Apoptosis (caspase-9/-3), cellular senescence, SASP	[3,55,73]
Oxidative Stress (Indirect)	Water radiolysis → ROS/RNS; mitochondrial ETC dysfunction	Cytochrome c → Apaf-1 → apoptosome → caspase-9/-3	Hepatocytes, mitochondria	Mitochondrial apoptosis, ferroptosis, lipid peroxidation	[68,69,70,71,72,74]
Cellular Senescence	Persistent DDR; failure of apoptosis	p53-mediated arrest; SASP: IL-6, IL-1β, TGF-β, TNF-α	Hepatocytes, HSCs	Chronic inflammation; activation of HSCs and immune cells	[75,76,77]
Inflammation	Kupffer cell & endothelial activation; ROS/NLRP3; SASP	NF-κB → TNF-α, IL-1β, IL-6; MAPK, JAK/STAT; ICAM-1/VCAM-1	Kupffer cells, endothelial cells, immune cells	Acute → chronic inflammation; recruitment of neutrophils/monocytes	[3,78,79,80,83]
Fibrosis	HSC activation; TGF-β release; bystander effect	TGF-β/Smad; PDGF; ECM deposition (collagen I/III)	HSCs, fibroblasts	Progressive fibrosis; ECM accumulation; liver dysfunction	[36,82]

## Data Availability

No new data were created or analyzed in this study. Data sharing is not applicable to this article.

## Supplementary Materials

The following supporting information can be downloaded at: https://www.mdpi.com/article/10.3390/jpm16090439/s1.
