# Supplementary material for: Structural and Functional Characteristics of the Liver After Fractionated Local Electron Irradiation and Against the Background of Ascorbic Acid Administration
Source: J Pers Med. 2026 Aug 22;16(9):439. doi: 10.3390/jpm16090439 (PMC13608792; doi:10.3390/jpm16090439)
Supplement: Supplementary file 1 [file jpm-16-00439-s001.zip › jpm-4417138-supplementary.pdf]

AI command/prompt used for Figure 1:

"Create a publication-quality scientific schematic comparing photon and electron irradiation for liver tumors. Show two side-by-side panels: (1) conventional photon irradiation using X-rays/γ-rays, with a photon beam entering the body, passing through the liver tumor and continuing beyond the target, illustrating entrance and exit doses and irradiation of surrounding healthy liver and adjacent organs at risk; and (2) electron irradiation using β-particles, with limited tissue penetration, concentration of the dose near the target, rapid dose fall-off after the treatment depth, and minimal exit dose. Accurately depict the relevant upper abdominal anatomy, including the liver in its normal anatomical position beneath the diaphragm, with the diaphragm clearly separating the thoracic and abdominal cavities. Show anatomically plausible adjacent structures, including the stomach and intestine, and illustrate greater sparing of deeper healthy tissues with electron irradiation compared with photon irradiation. Indicate the approximate penetration depth of conventional electron irradiation (~2–3 cm) and the concept of rapid dose fall-off. Emphasize the dosimetric difference between photon and electron irradiation rather than depicting a specific clinical treatment plan. Electron beams are considered promising for superficial liver tumors, intraoperative radiotherapy (IORT), FLASH radiotherapy, and VHEE applications. Use clear scientific labels for photon irradiation (X-rays/γ-rays), electron irradiation (β-particles), entrance dose, exit dose, limited penetration, and rapid dose fall-off. Use a clean, professional biomedical illustration style suitable for a peer-reviewed scientific journal, with no decorative elements, tables, or unnecessary text".

AI command/prompt used for Figure 2:

"Create a publication-quality scientific schematic illustrating the pathogenesis of radiation-induced liver disease (RILD) following electron irradiation. Show two major mechanisms: (1) direct radiation-induced DNA damage leading to SSB/DSB, ATM/ATR–p53 activation, apoptosis and cellular senescence/SASP; and (2) indirect radiation effects through water radiolysis, ROS/RNS generation, oxidative stress, mitochondrial dysfunction, cytochrome c release, caspase activation, apoptosis and ferroptosis. Integrate the effects on hepatocytes, sinusoidal endothelial cells, hepatic stellate cells, Kupffer cells and infiltrating immune cells. Show endothelial dysfunction and microvascular injury, inflammatory signaling through NF-κB and pro-inflammatory cytokines, and activation of hepatic stellate cells through TGF-β and related profibrotic pathways. Connect these mechanisms to extracellular matrix deposition, collagen accumulation, fibrosis and ultimately radiation-induced liver disease with hepatocellular injury and impaired liver function".

It should be noted that the final figures were not produced from a single prompt. The AI-generated outputs were iteratively refined through multiple prompts, and the resulting drafts were then substantially edited in Adobe Photoshop — including repositioning of structures, rewriting and repositioning labels, adding arrows and connectors, and adjusting colors and layout to achieve the final publication-ready versions. The authors critically reviewed and validated every element of the figures. The final figures therefore represent a collaborative process between the authors and the AI tool, with the authors exercising full editorial control.
